# High Proton-Conductive and Temperature-Tolerant PVC-P4VP Membranes towards Medium-Temperature Water Electrolysis

**DOI:** 10.3390/membranes12040363

**Published:** 2022-03-25

**Authors:** Yichen Yin, Yiming Ying, Guojuan Liu, Huiling Chen, Jingrui Fan, Zhi Li, Chuhao Wang, Zhuangyan Guo, Gaofeng Zeng

**Affiliations:** 1 CAS Key Laboratory of Low-Carbon Conversion Science and Engineering, Shanghai Advanced Research Institute, Chinese Academy of Sciences, Shanghai 201210, China; yinyichen2019@sari.ac.cn (Y.Y.); yingym@shanghaitech.edu.cn (Y.Y.); liuguoj@sari.ac.cn (G.L.); chenhl@sari.ac.cn (H.C.); fanjr@sari.ac.cn (J.F.); lizhi2@shanghaitech.edu.cn (Z.L.); wangchh3@shanghaitech.edu.cn (C.W.); guozhy@shanghaitech.edu.cn (Z.G.); 2 School of Chemical Engineering, University of Chinese Academy of Sciences, Beijing 100049, China; 3 School of Physical Science and Technology, ShanghaiTech University, Shanghai 201210, China

**Keywords:** proton exchange membrane, polyvinyl chloride/poly(4vinylpyridine) membrane, cross-link, proton conductivity, high-temperature water electrolysis

## Abstract

Water electrolysis (WE) is a highly promising approach to producing clean hydrogen. Medium-temperature WE (100–350 °C) can improve the energy efficiency and utilize the low-grade water vapor. Therefore, a high-temperature proton-conductive membrane is desirable to realize the medium-temperature WE. Here, we present a polyvinyl chloride (PVC)-poly(4vinylpyridine) (P4VP) hybrid membrane by a simple cross-linking of PVC and P4VP. The pyridine groups of P4VP promote the loading rate of phosphoric acid, which delivers the proton conductivity of the PVC-P4VP membrane. The optimized PVC-P4VP membrane with a 1:2 content ratio offers the maximum proton conductivity of 4.3 × 10^−2^ S cm^−1^ at 180 °C and a reliable conductivity stability in 200 h at 160 °C. The PVC-P4VP membrane electrode is covered by an IrO_2_ anode, and a Pt/C cathode delivers not only the high water electrolytic reactivity at 100–180 °C but also the stable WE stability at 180 °C.

## 1. Introduction

For the global carbon-neutral vision and clean energy, hydrogen (H_2_) is considered as the essential alternative [1,2,3,4,5,6,7]. To reduce the carbon footprint in the production of hydrogen, water electrolysis (WE), which combines with the renewable powers generated from solar and wind, is an inevitable choice to replace the current fossil-fuel-derived H_2_ [8]. According to the operation temperature, the water electrolysis can be divided into three phases that conduct at low temperatures (<100 °C), medium temperatures (100–400 °C) and high temperatures (>600 °C), respectively [9,10]. The low-temperature WE is widely investigated through either the OH^-^ conductive membrane electrodes or the proton-conductive membrane electrodes [9,10]. At the same time, the high-temperature steam electrolysis based on O^2−^ conductive solid oxides is developed to reduce the electrical energy consumption [11]. However, there is not enough research on the medium-temperature water electrolysis. Similar to the high-temperature water electrolysis, the medium-temperature water electrolysis is also expected to save electrolytic power by utilization of the heat of the water, in which the molar Gibbs energy of the reaction drops ~12% at 250 °C compared to the room temperature [12], since the steam at 100–350 °C is a low-grade waste heat in various industries [12]. Moreover, medium-temperature water electrolysis is a potential method to make the waste heat into a usable energy.

Both the current low-temperature and high-temperature ion-conductive membranes cannot meet the requests of medium-temperature water electrolysis. For the former, commercial proton exchange membranes, such as the Nafion membrane, cannot withstand temperatures higher than 100 °C for a long time [13]. For the latter, the solid oxide membranes are non-ion-conductive at 100–350 °C [14]. To enhance the thermal stability of polymer membranes, phosphoric-acid-doped polybenzimidazole (PA/PBI) membranes are widely investigated [15]. However, the high cost of PBI limits the application of these membranes [16]. Polyvinyl chloride (PVC) is currently the second-largest resin in the world, which takes the advantages of high thermal stability and low cost and thus is widely used in industry, agriculture and plastic building materials [17]. By introducing nitrogen-containing groups into the PVC matrix for acid adsorption, the PVC-based membranes can obtain proton conductivity [18]. For the adsorption of acid, various N-heterocyclic groups (such as piperidine, pyridine, pyrrolidone [19] and quaternary ammonium [20], tertiary amine groups [21] and imidazolium [22,23]) have been introduced into the polymer side chain. 4-vinylpyridine (4VP) is an important functional monomer with a pyridine group, which is used to graft onto other polymers to prepare proton exchange membranes [24,25,26]. On the other hand, poly 4-vinylpyridine (P4VP) cannot be used as a proton exchange membrane alone because it is unstable in the PA doping condition [25]. Therefore, the combination of PVC and P4VP is reasonable to obtain not only thermal stability but also high proton conductivity.

In this work, PVC was cross-linked with P4VP to form PVC-P4VP membranes. With the adsorption of phosphoric acid, the resultant membranes exhibit proton conductivities at temperatures up to 180 °C, which were then used for the medium-temperature water electrolysis. Fourier transform infrared spectroscopy (FTIR), X-ray photoelectron spectroscopy (XPS) and X-ray diffraction (XRD) were employed to prove the cross-linked structure. The mechanical properties and ionic conductivity of the high-temperature proton exchange membranes were also investigated in detail.

## 2. Materials and Methods

### 2.1. Materials

Ethanol (99.7%), isopropanol (99.7%), sulfuric acid (95.0~98.0%), N, N-dimethylacetamide (DMAc, 99.0%), H_2_O_2_ (30%) and iron (II) sulfate heptahydrate (99.0~101.0%) were purchased from Sinopharm Chemical Reagent Co. (Shanghai, China). Polyvinyl chloride (PVC), Poly(4-vinyl pyridine) (P4VP) with an average molecular weight of 16,000, iridium oxide (99.9% metals basis, Ir ≥ 84.5%) and orthophosphoric acid (85% in water) were purchased from Shanghai Titan Technology Co (Shanghai, China). Nafion solution (5 wt.% in mixture of lower aliphatic alcohols and water, contains 45% water), Pt/C catalyst (JM, 40 wt.% Pt) and carbon paper were purchased from Shanghai Hesen Electric Co (Shanghai, China). All the materials were used as received without further treatments.

### 2.2. Membrane Preparation

P4VP (0.5 g) and PVC with various weight ratios of 0:1, 1:1, 1.5:1 and 2:1 were dissolved in DMAc at room temperature by stirring. The cross-link reaction between PVC and P4VP was conducted at 130 °C for 3 h. The DMAc solution of cross-linked PVC-P4VP was degassed in vacuum for 30 min before membrane preparation. The membranes were formed by tape-casting method on a clean glass plate at room temperature. Then, the glass plate was heated to 80 °C for 2 h for solidification and solvent evaporation. The as-prepared membranes were immersed in 85 wt.% phosphoric acid (PA) aqueous solution for 24 h to load phosphoric acid.

The PA doping mass ratio was calculated by measuring the weight difference of membranes before and after PA treatment, as shown in Equation (1) [15]:

(1) $$\mathrm{PA}\;\mathrm{doping}\;\mathrm{mass}\;\mathrm{ratio}=\frac{W_{A}-W_{B}}{W_{B}}$$

where *W*_B_ and *W*_A_ are the membrane weights before and after phosphoric acid treatment, respectively. Chemical stability of membranes was tested by immersing the membrane samples in Fenton’s reagent (H_2_O_2_, 3 wt.%; Fe^2+^, 4 ppm) at 68 °C. The treated membranes were then dried and weighted (Table S1).

### 2.3. Water Electrolysis over PVC-P4VP/PA Membrane

The conductivity of membranes was measured by the alternating current impedance method (IMP) using an electrochemical workstation (CHI 760E, Shanghai C&H, Shanghai, China) with a frequency range of 100–100,000 Hz [15]. The conductivity (σ, S m^−1^) was calculated according to Equation (2):

(2) $$\sigma =\frac{w}{S*R}$$

where *w* (m) is the thickness of membranes, *S* is the surface area of membranes (m^2^) and *R* is the measured impedance (Ω). 

For the water electrolysis, IrO_2_ and Pt/C were the anode and cathode catalysts, respectively [27]. Isopropyl alcohol and ethanol were used as the organic solvents in the preparation of the catalyst slurry. In total, 5% Nafion solution was added to increase the interactions between catalyst and membrane. In the experiment, the capacity of anode catalyst powder in the membrane electrode was controlled to be 2.5 mg cm^−2^, and the ratio of anode catalyst Pt/C and IrO_2_ was 2:8. The loading rate of cathode catalyst was 0.3 mg cm^−2^. Considering the adhesion of catalytic layer, the ratio of dry Nafion addition to catalyst was 1:1. The catalyst slurry was sprayed onto the membrane surface, which was then dried at room temperature. The membrane electrode was covered with carbon papers (HCP020P) by using the hot-pressing method. Linear scanning voltammetry (LSV) and current time curves (*i-t*) were performed using a CHI 760E (Shanghai C&H) electrochemical workstation at the temperature range of 100–180 °C. 

### 2.4. Characterizations

The morphology of membranes was measured by a scanning electron microscope (SEM, SUPRA 55 SAPPHIRE Carl Zeiss, Oberkochen, Germany). The structure of membranes was tested by using a Rigaku Ultima IV X-ray powder diffractometer (XRD) with a Cu Kα target (λ = 1.54056 Å) at a scanning speed of 5° min^−1^ from 10 to 65° (40 kV and 40 mA). The chemistry of membrane samples was analyzed by a Fourier transform infrared spectrometer (FTIR, Thermo Scientific, Nicolet Magna 550, Waltham, MA, USA) in the scanning range of 400–4000 cm^−1^ with a resolution frequency of 2 cm^−1^ for 64 times scan. X-ray photoelectron spectroscopy (XPS) measurements were carried out on a Thermo Scientific K-Alpha XPS spectrometer using Al Kα X-ray source for radiation. The mechanical properties of membranes were characterized by using a CMT6103 electronic universal test machine from MTS at room temperature at a rate of 2 mm min^−1^. The thermal stability of PVC powder, PVC-P4VP and PVC-P4VP/PA membranes was measured using a thermogravimetric analyzer (TGA, STA449F3, Netzsch, Selb, Germany) with a heating rate of 10 °C min^−1^ in nitrogen.

## 3. Results and Discussion

### 3.1. Synthesis of PVC-P4VP/PA Membranes through Cross-Linking

**Figure 1:**
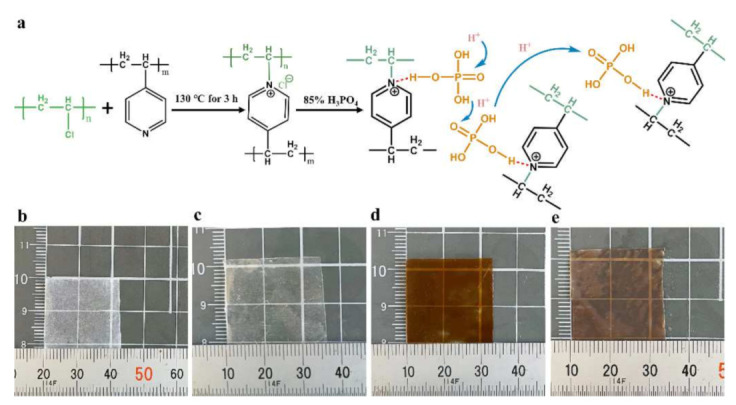
(**a**) Schematic of the preparation of PVC-P4VP/PA membrane through cross-linking reaction and the optical images of PVC (**b**), P4VP (**c**), PVC-P4VP (**d**) and PVC-P4VP/PA (**e**) membranes.

The synthesis of the PA-doped PVC-P4VP membrane (PVC-P4VP/PA) involves two steps. Firstly, PVC and P4VP were cross-linked in solvothermal conditions [28,29]. Then, the cross-linked PVC-P4VP membrane was doped with PA through the hydrogen bonds between PA and rich pyridinium groups (Figure 1a). The doped PA molecules provide transfer paths for protons in the applications [24,25,26]. During these steps, the membrane color changes from transparent colorless PVC and P4VP precursors to brown for the PVC-P4VP and light brown for the PVC-P4VP/PA membrane (Figure 1b–e). This indicates that the reaction between PVA and P4VP was completed and PA was introduced into PVC-P4VP, which are further confirmed by characterization hereinafter. 

**Figure 2:**
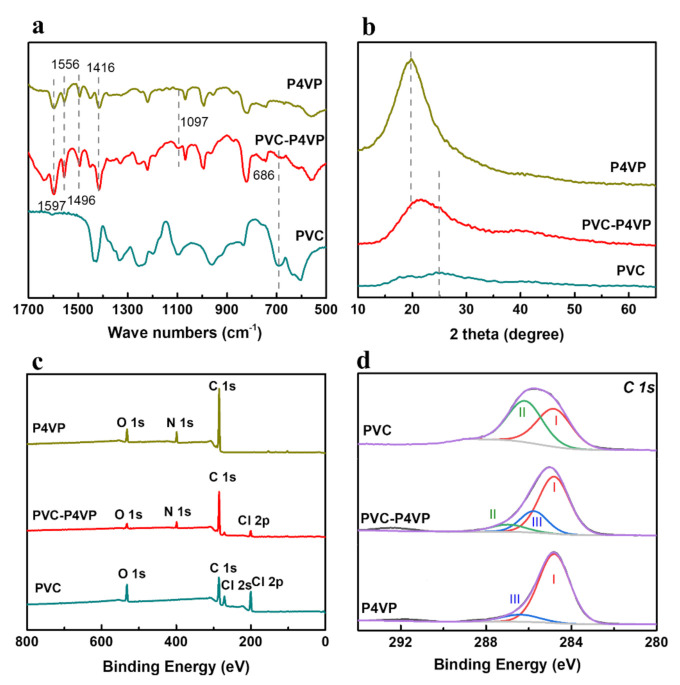
(**a**) FTIR, (**b**) XRD patterns, (**c**) XPS survey spectra and (**d**) C 1s spectra of PVC, P4VP and PVC-P4VP samples.

As shown in Figure 2a, the FTIR spectrum of PVC showed a stretching vibration of C-Cl at 686 cm^−1^ [30], while it was significantly decreased in the spectrum of PVC-P4VP. This indicates that most of the C-Cl groups are consumed and substituted in the cross-linking reaction. At the same time, the absorption signals at 1416 cm^−1^ and 1496 cm^−1^ in the FTIR spectrum of P4VP are assigned to the C=C stretching vibration in the pyridine ring, and the bands at 1556 cm^−1^ and 1597 cm^−1^ are ascribed to the C=N/C-N stretching vibration in the pyridine ring [31,32], respectively, which are well retained in the spectrum of PVC-P4VP. This proves that P4VP was successfully introduced into the PVC matrix. Moreover, a new band at 1097 cm^−1^ [3], being contributed to the out-of-plane bending vibration of C-N, was observed from PVC-P4VP. This directly confirms the successfully cross-linking of PVC and P4VP via replacing -Cl of C-Cl with pyridine N. The XRD patterns present a broad peak at 2θ = ~25° for the PVC membrane [33] and a strong signal at 2θ = ~19° for the P4VP membrane [34], which are attributed to the ordered matrix of PVC and P4VP (Figure 2b). In contrast, the peak of the PVC-P4VP membrane shifts to 2θ = ~22°, located between PVC and P4VP. This reveals that the PVC-P4VP still keeps an ordered structure after the cross-linking reaction.

The elemental composition and surface chemistry of PVC, P4VP and PVC-P4VP were investigated by XPS. The XPS survey spectrum of PVC-P4VP displays a new N signal compared to that of PVC, confirming the introduction of P4VP in the cross-linking reaction (Figure 2c). At the same time, the content of Cl in PVC-P4VP decreases significantly from 19.1% of PVC to 9.2%. On one hand, the cross-linked P4VP dilutes the Cl concentration on the PVC-P4VP surface. On the other hand, it also indicates that the C-Cl groups are replaced by the pyridine group in the cross-linking reaction, which is consistent with the FTIR observations. The C 1s spectra can be deconvoluted into three peaks centered at 284.8 eV (C-C bond), 286.3 eV (C-Cl bond) and 285.6 eV (C-N bond), as shown in Figure 2d [35]. For PVC-P4VP, the proportion of the C-Cl bond decreased from 51.2% of the PVC to 9.6%, in line with the former results. Meanwhile, the spectrum of N 1s delivers a small peak of NH^+^ (400.59 eV) [35,36], apart from the main peak of N*-C (398.8 eV), as shown in Figure S1. Correspondingly, the Cl 2p of PVC-P4VP presents an inorganic Cl^-^ signal at 197.7 eV, in addition to organic chloride at 200.0 eV and 201.7 eV (Figure S2) [37], which keeps the charge balance of the PVC-P4VP illustrated in Figure 1a.

**Figure 3:**
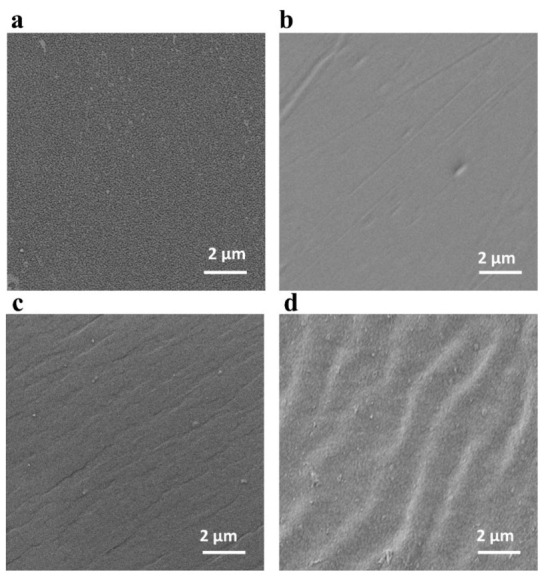
SEM surface images of PVC (**a**), P4VP (**b**), PVC-P4VP (**c**) and PVC-P4VP/PA membranes (**d**).

The morphology of the membranes was measured by SEM. Before cross-linking, both the PVC membrane and the P4VP membrane display dense and smooth surfaces (Figure 3a,b). This reveals the excellent flexibility of these precursors for membrane preparation. After cross-linking, the PVC-P4VP membrane retains a smooth surface (Figure 3c). Upon the immersion of phosphoric acid, the surface of the PVC-P4VP/PA presents wrinkles. As the PA was introduced into the matrix of the PVC-P4VP, the hybrid membrane swelled to some extent, which caused the deformation of membrane morphology. This is consistent with that of the PBI-NH_2_-EPA-15 membrane [38]. Correspondingly, the cross-section views of PVC, P4VP and PVC-P4VP also show dense and uniform morphology (Figures S3–S5). The cross-section of PVC-P4VP posts visible wrinkles with a pore structure, in line with the surface observations (Figure S6). 

### 3.2. Thermal/Mechanical/Chemical Stability of PVC-P4VP/PA Membranes

**Figure 4:**
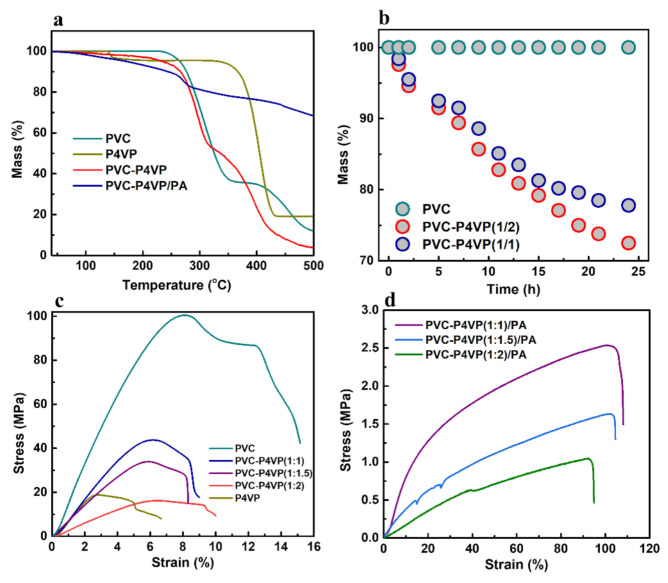
(**a**) TGA curves of PVC, P4VP, PVC-P4VP and PVC-P4VP/PA membranes in N_2_, (**b**) mass changes of PVC, PVC-P4VP(1/2) and PVC-P4VP(1/1) immersed in Fenton’s reagent at 68 °C, tensile stress-strain tests of (**c**) PVC, P4VP, PVC-P4VP(1:1), PVC-P4VP(1:1.5) and PVC-P4VP(1:2) membranes and (**d**) PVC-P4VP(1:1)/PA, PVC-P4VP(1:1.5)/PA and PVC-P4VP(1:2)/PA membranes.

The thermal properties of the membranes were measured by TGA in N_2_. As shown in Figure 4a, the PVC remains stable at temperatures below 250 °C and then two mass loss phases occur at ~260 °C and 420 °C due to the loss of HCl and the decomposition of the PVC matrix, respectively [30]. Apart from a samll amount of water molecule loss at low temperatures of ~150 °C [39], the P4VP sample shows a main mass loss at 380 °C. Therefore, both precursors exhibit high thermal stability. Similar to P4VP, the PVC-P4VP sample shows slight mass loss from 100 to 270 °C, which is assigned to water loss. This suggests that the PVC-P4VP membrane is temperature tolerant at the target temperature [40]. The main mass loss of the PVC-P4VP membrane starts at ~250 °C, which is ascribed to the loss of unreacted -Cl groups and then the decomposition of the PVC matrix. At the same time, PVC-P4VP exhibits a slightly higher mass loss rate than that of both precursors at high temperatures ranging from 370 °C to 500 °C. It is reasonable that the cross-link reaction slightly declines the bonding strength and thus impacts the stability at very high temperatures, which was also observed in the previously reported works [41,42]. The PVC-P4VP/PA membrane shows a continuous mass loss during the TGA measurement, resulting in 10% mass loss before 280 °C. The evaporation of water molecules in the dopped PA phase may contribute to the mass loss of PVC-P4VP/PA in the low temperature region of 100–280 °C. Compared to the PVA-P4VP membrane that only remained at ~4% residual weight at 500 °C, the high residual weight (~69%) of PVC-P4VP/PA indicates the high doping amount of PA, which would dehydrate to P_2_O_5_ at high temperatures [43]. 

The Fenton reagent is commonly used to test the chemical stability of membrane samples because it can offer strong oxidation conditions with H_2_O_2_ and Fe^2 +^ [44]. No visible mass loss of the PVC membrane is observed in the Fenton reagent at 68 °C for 24 h, which reveals a high antioxidation chemical stability of PVC (Figure 4b). At the same time, the PVC-P4VP (1/1) and the PVC-P4VP (1/2) membranes lost about 22% and 28% weight, respectively, under the same conditions (Figure 4b). Since the chemical stability of PVC has been demonstrated, the weight loss is attributed to the decomposition of P4VP. However, the PVC-P4VP membrane still exhibited reliable chemical stability compared to the previously reported membranes. For examples, the ionic liquid-doped sulphonated polyether ether ketone (SPEEK) membranes showed 40–50% mass loss under the same conditions [45], and the mass loss of the PVC membrane doped with methylimidazolium groups (phosphoric acid) took a 40% mass loss in the Fenton reagent treatments [17]. In addition, unlike the reported membrane that generated bubbling on the membrane surface, the PVC-P4VP membrane received no changes on the integrity of the membrane during the Fenton reagent treatment for 24 h [46]. This confirms the chemical stability of PVC-P4VP upon oxidation conditions.

The mechanical properties of the membranes were studied, as shown in Figure 4c,d. The PVC membrane displays a nonlinear elastic deformation region from 0 to 8% and a plastic deformation region from 8 to 15%. In comparison, the P4VP membrane posts an elastic deformation from 0 to 2.5% and a plastic deformation from 2.5% to 7%. Therefore, the cross-linked PVC-P4VP membranes exhibit an elastic deformation region between that of PVC and P4VP (Figure 4c). After introducing PA, the PVC-P4VP/PA membranes show significantly enhanced nonlinear elastic deformation with a tensile stress of 1.04–2.51 MPa at the tensile strains of 93–104% (Table S2). This is comparable to the reported membranes, such as PVC-MIMCI/PA (1 MPa) [17]. 

### 3.3. Electrochemical Properties and Electrolytic Water Performance

**Figure 5:**
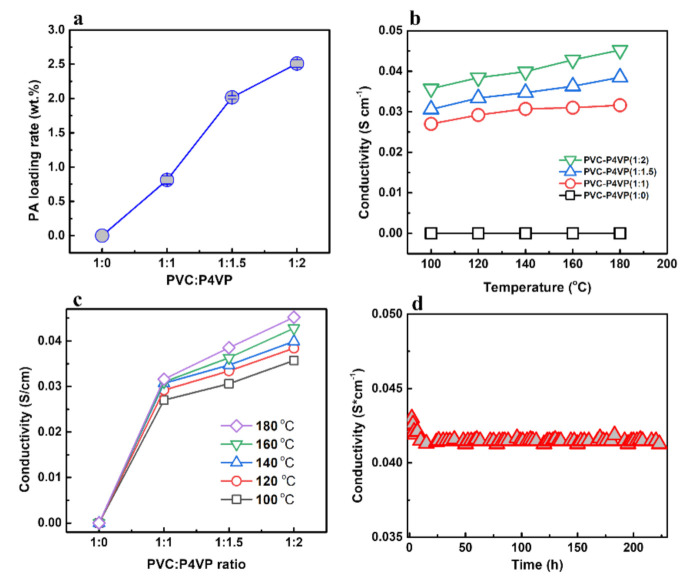
(**a**) Phosphoric acid doping rate of PVC-P4VP (1:X) after immersion in concentrated phosphoric acid, (**b**) temperature dependence of proton conductivity on PVC-P4VP (1:X)/PA membranes, (**c**) effects of PVC/P4VP ratio on the proton conductivity at different temperatures and (**d**) stability of proton conductivity of PVC-P4VP (1:2)/PA membrane at 160 °C.

Since PA is the transport medium for protons, the proton conductivity of the membranes depends on the PA density in the membranes. As shown in Figure 5a, the PA adsorption rate increases with the introduction of P4VP, starting from 0 of PVC to 0.8 wt.% of PVC-P4VP (1:1), 2.1 wt.% of PVC-P4VP (1:1.5) and further to 2.6wt.% of PVC-P4VP (1:2). It is reasonable that the introduced N-groups of P4VP contribute to the adsorption capacity of the membranes. However, the membranes would become brittle with the introduction of more P4VP. 

The proton conductivity of the membrane was calculated with the measured impedance by using Equation (2) (Figure S7). Figure 5b shows the effect of temperature on the proton conductivity of the PVC-P4VP(1:X)/PA membranes. In the temperature range of 100–180 °C, no proton conductivity was detected for the PVC membranes because there is no proton transfer media in PVC. In comparison, all PVC-P4VP membranes are proton-conductive. The conductivity of PVC-P4VP(1:2)/PA increases from 3.5 × 10^−2^ S cm^−1^ at 100 °C to 4.5 × 10^−2^ S cm^−1^ at 180 °C because the high temperature promotes the proton transfer. On the other hand, the proton conductivity increased with the increase of P4VP content in the membrane (Figure 5c). The proton conductivity increases from 3.1 × 10^−2^ S cm^−1^ for PVC-P4VP(1:1)/PA to 3.8 × 10^−2^ S cm^−1^ for PVC-P4VP(1:1.5)/PA and 4.5 × 10^−2^ S cm^−1^ for PVC-P4VP(1:2)/PA. With increased P4VP cross-linking, P4VP provides more pyridine sites and thus more PA can be adsorbed on the membrane. As a result, more proton transfer paths lead to the improved conductivity of the membranes with high PA doping levels. Compared with the reported PBI/P4VP (50/50) (W/W), PVPA/P4VP-NS blends and ETFE-g-P4VP membranes, the PVC-P4VP(1:2)/PA membrane has superior proton conductivity (Table 1).

The proton-transport activation energy was determined by the Arrhenius equation (Equation (3)) [48]:

(3) $$\ln\left(\sigma T\right)=lnA-\frac{Ea}{k_{B}T}\;$$

where the symbols σ, *A*, *Ea* and *k_B_* represent the proton conductivity, pre-exponential factor, proton transport activation energy and Boltzmann constant, respectively. The activation energy values were 12.0 KJ mol^−1^ (PVC-P4VP(1:1)/PA), 11.5 KJ mol^−1^ (PVC-P4VP(1:1.5)/PA) and 10.8 KJ mol^−1^ (PVC-P4VP(1:2)/PA) in the temperature range of 100–180 °C (Figure S8). The Ea values decrease with the increase of P4VP, indicating that the introduction of P4VP reduces the resistance of the proton transfer. As the reported Ea of the proton transfer in 85% PA is 14.3 KJ mol^−1^ [49], this reveals that the proton transport behavior in the membrane is similar to that in the concentrated phosphoric acid.

The effects of the P4VP doping ratio on the proton conductivity were shown in Figure 5c. At the same temperature, the proton conductivity of the membrane gradually increases with the increase of P4VP content in PVC-P4VP because more P4VP can fix more PA and thus contribute to higher proton transfer capacity. The long-term stability of the proton conductivity of the PVC-P4VP(1:2)/PA membrane was subsequently tested at 160 °C under water-free conditions (Figure 5d). The conductivity rapidly decreased from 4.23 × 10^−2^ S cm^−1^ to 4.15 × 10^−2^ S cm^−1^ during the first 10 h, and then it kept stable at 4.15 × 10^−2^ S cm^−1^ for 220 h. Similarly, the PVC-P4VP(1:2)/PA membrane also presents stable conductivity around 3.85 × 10^−2^ S cm^−1^ at 140 °C for 210 h (Figure S9). Thus, it was confirmed that the PVC-P4VP(1:2)/PA has enough thermal and chemical stability for high-temperature applications.

**Figure 6:**
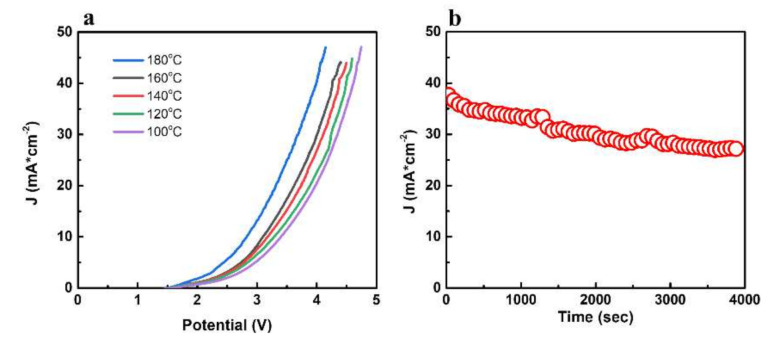
(**a**) Polarization curves of electrolysis cells using PVC-P4VP (1:2)/PA membranes at different temperatures and (**b**) stability of electrolysis cells using PVC-P4VP (1:2)/PA membranes at 180 °C.

The PVC-P4VP(1:2)/PA membrane electrode was assembled into a water electrolysis cell. Figure 6a shows the polarization curves of the PVC-P4VP(1:2)/PA membrane electrode at 100–180 °C with an interval of 20 °C. The current density increases with the increasing temperature for a certain cell voltage. At 180 °C, the current density changes rapidly with increasing voltage, which is attributed to the enhanced electrocatalytic activity and the decrease in cell ohmic resistance at high temperatures. With the cell voltage of 3 V, the current density at 180 °C (16.1 mA cm^−2^) is more than three times higher than that at 100 °C (5.2 mA cm^−2^). This indicates the superior performance of the PVC-P4VP (1:2)/PA membrane because that current density depends on the conductivity of the membrane, the electrolyte concentration, the membrane thickness and the membrane resistance. The stability of the membrane electrode was studied at 180 °C with a constant voltage of 3.5 V (Figure 6b). The current density slightly decreased from 38 mA cm^−2^ to 30 mA cm^−2^ after 1 h. This indicates that the phosphoric-acid-doped PVC-P4VP(1:2)/PA membranes exhibit good stability in a water electrolysis environment at high temperatures.

## 4. Conclusions

In summary, the phosphoric-acid-doped PVC-P4VP membranes that possess high proton conductivity at high temperatures were achieved by a simple cross-linking between PVC and P4VP. With this strategy, the pyridine groups of P4VP react with the C-Cl group of PVC to form a new polymeric matrix with C-N connections. The proton conductivity of the PVC-P4VP membrane depends on the P4VP content, since the pyridine groups of P4VP provide interaction sites for the adsorption and immobilization of PA, thus improving the proton transfer capability of the membrane. The optimized PVC-P4VP content ratio of 1:2 exhibited a maximum proton conductivity of 4.5 × 10^−2^ S cm^−1^ at 180 °C and a long-term conductivity stability of 200 h at 160 °C. The PVC-P4VP membrane electrode with a commercial IrO_2_ anode and Pt/C cathode had high water electrolytic reactivity in the range of 100–180 °C.

## Figures and Tables

**Table 1:** Comparison of proton conductivity of PVC-P4VP(1:2)/PA membrane with reported membranes at high temperature.

Membrane Material	Conductivity (10^−2^ S cm^−1^)	Temperature (°C)	References
PBI/P4VP (50/50) (W/W)	1.8	140	[47]
PVPA/P4VP-NS blends	0.02	140	[28]
PVC-P4VP(1:2)/PA	4.0	140	This work
ETFE-g-4VP (0% DVB 30%GL)	2.8	120	[42]
ETFE-g-P4VP	4.0	120	[43]
PVC-P4VP(1:2)/PA	3.8	120	This work

## Data Availability

Not applicable.

## Supplementary Materials

The following supporting information can be downloaded at: https://www.mdpi.com/article/10.3390/membranes12040363/s1, Figure S1: XPS N 1s spectra of PVC, PVC-P4VP and P4VP samples; Figure S2: XPS Cl 2p spectra of PVC, PVC-P4VP and P4VP samples; Figure S3: SEM cross-sectional view of PVC membrane; Figure S4: SEM cross-sectional view of P4VP membrane; Figure S5: SEM cross-sectional view of PVC-P4VP membrane; Figure S6: SEM cross-sectional view of PVC-P4VP/PA membrane; Figure S7: Nyquist plots of (a) PVC-P4VP(1:1)/PA, (b) PVC-P4VP (1:1.5)/PA, (c) PVC-P4VP(1:2)/PA at 100–180 °C; Figure S8: Temperature dependence of conductivity with PVC-P4VP membranes; Figure S9: Stability of proton conductivity of PVC-P4VP(1:2)/PA membrane at 140 °C; Table S1: The thickness and surface area of membranes; Table S2: Stress-strain parameter for PVC, P4VP, PVC-P4VP(1/X) and PVC-P4VP(1/X)/PA membranes.
