# Supplementary material for: High Proton-Conductive and Temperature-Tolerant PVC-P4VP Membranes towards Medium-Temperature Water Electrolysis
Source: Membranes (Basel). 2022 Mar 25;12(4):363. doi: 10.3390/membranes12040363 (PMC9027779; doi:10.3390/membranes12040363)
Supplement: Supplementary file 1 [file membranes-12-00363-s001.zip › membranes-1646062-supplementary.pdf]

membranes

Supporting information

High Proton-Conductive and Temperature-Tolerant PVC-P4VP Membranes Towards Medium-Temperature Water Electrolysis

Yichen Yin[1,2], Yiming Ying[1,3], Guojuan Liu[1,2], Huiling Chen[1,2], Jingrui Fan[1,2], Zhi Li[1,3], Chuhao Wang[1,3], Zhuangyan Guo[1,3] and Gaofeng Zeng [1,2,] *

1 CAS Key Laboratory of Low-carbon Conversion Science and Engineering, Shanghai Advanced Research Institute, Chinese Academy of Sciences, Shanghai 201210, China
2 School of Chemical Engineering, University of Chinese Academy of Sciences, Beijing 100049, China
3 School of Physical Science and Technology, ShanghaiTech University, Shanghai 201210, China
* Correspondence: zenggf@sari.ac.cn

Figures

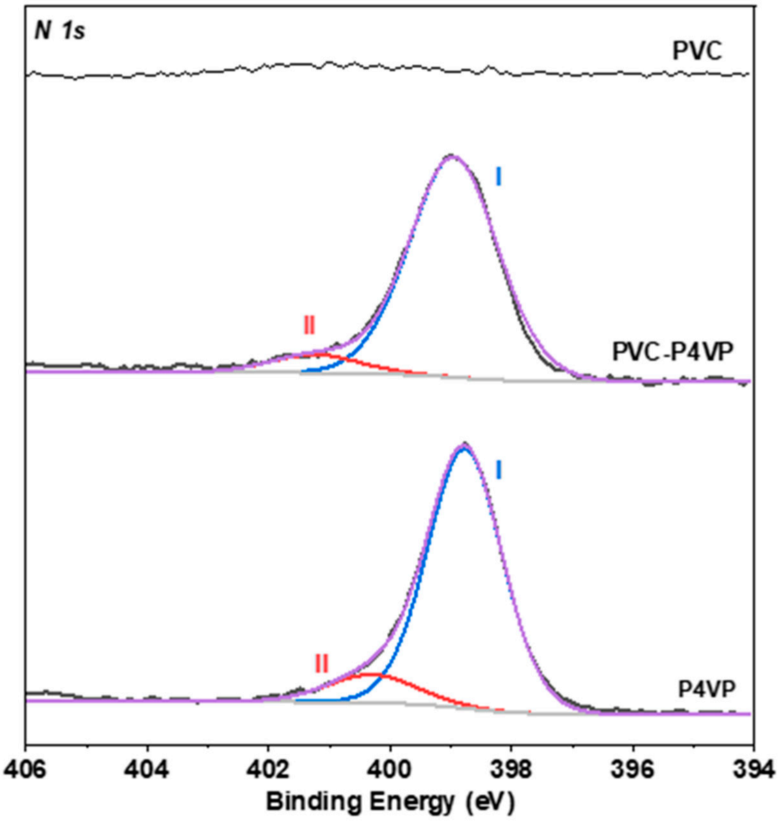

Figure S1. XPS N 1s spectra of PVC, PVC-P4VP and P4VP samples.

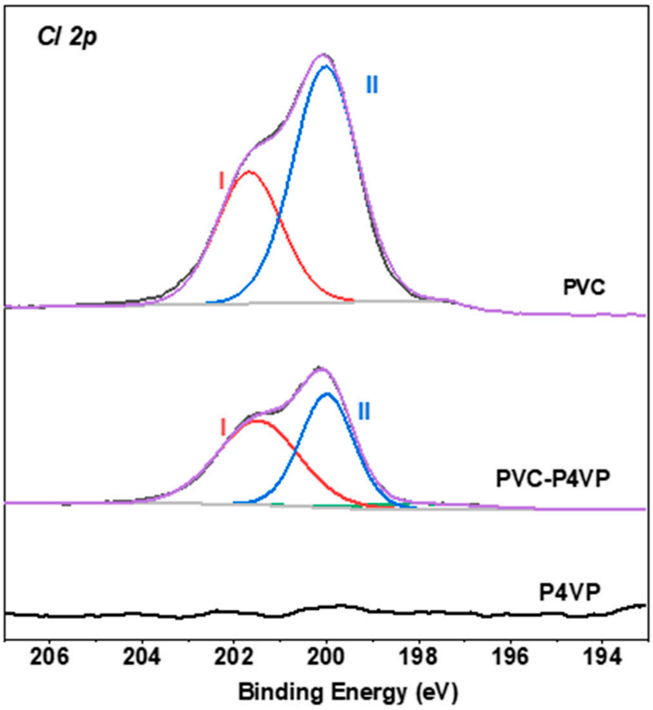

Figure S2. XPS Cl 2p spectra of PVC, PVC-P4VP and P4VP samples.

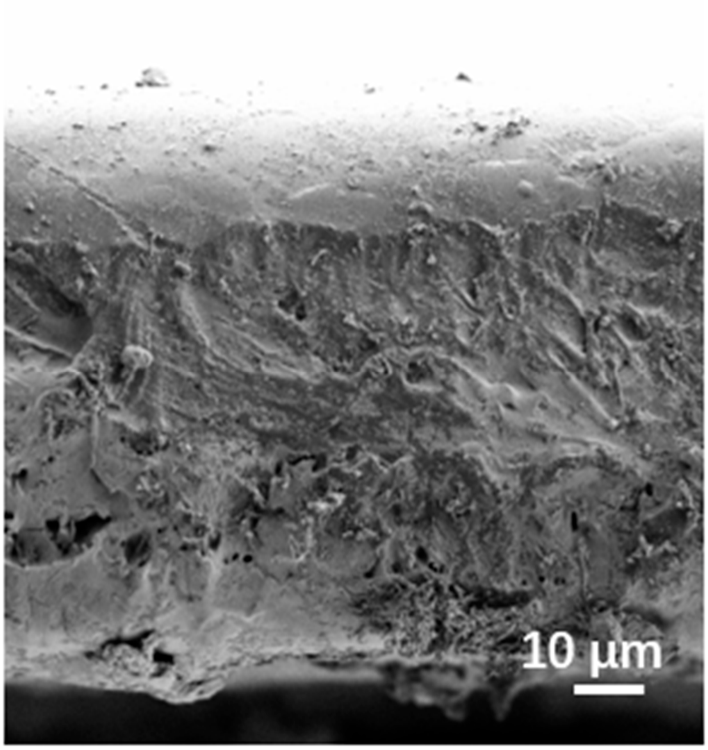

Figure S3. SEM cross-sectional view of PVC membrane.

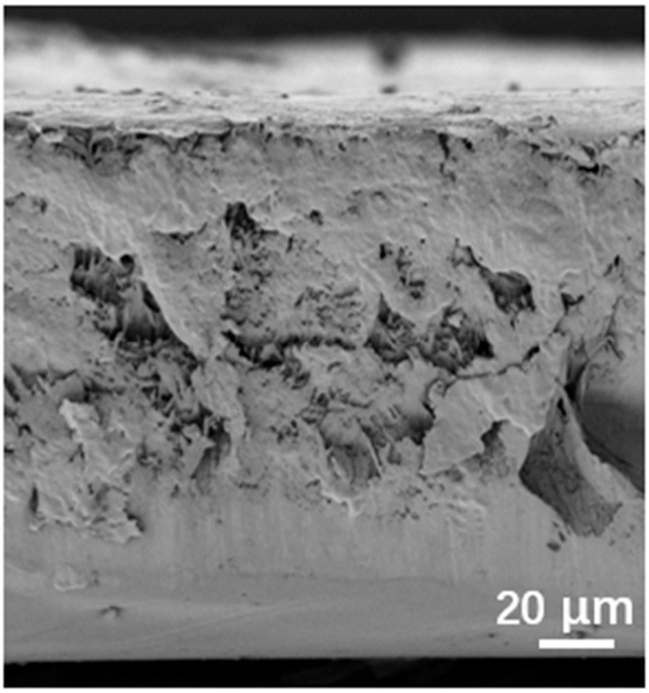

Figure S4. SEM cross-sectional view of P4VP membrane.

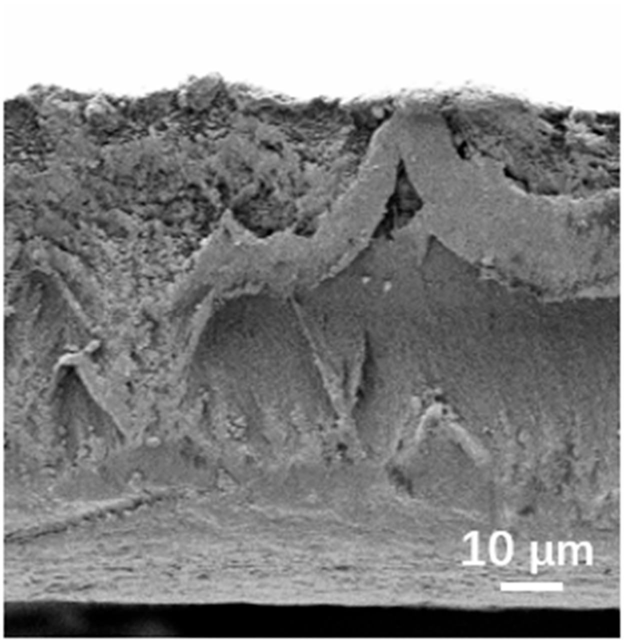

Figure S5. SEM cross-sectional view of PVC-P4VP membrane.

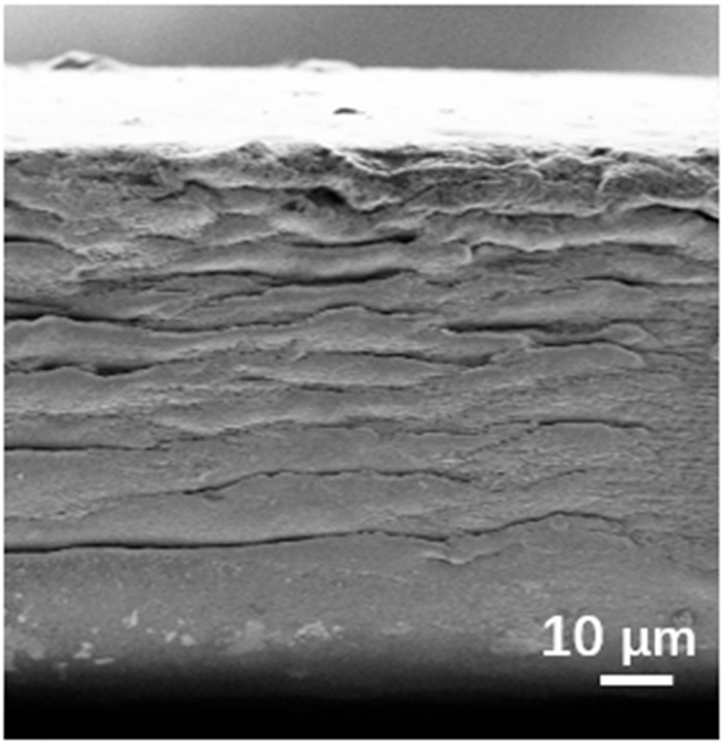

Figure S6. SEM cross-sectional view of PVC-P4VP/PA membrane.

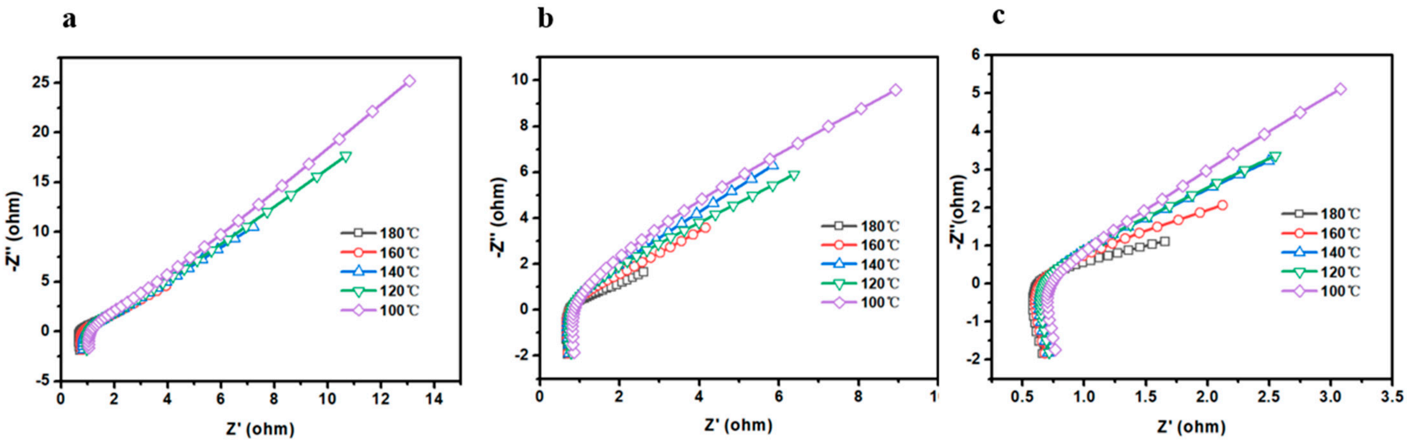

Figure S7. Nyquist plots of (a) PVC-P4VP(1:1)/PA, (b) PVC-P4VP (1:1.5)/PA , (c) PVC-P4VP(1:2)/PA at 100-180 ℃.

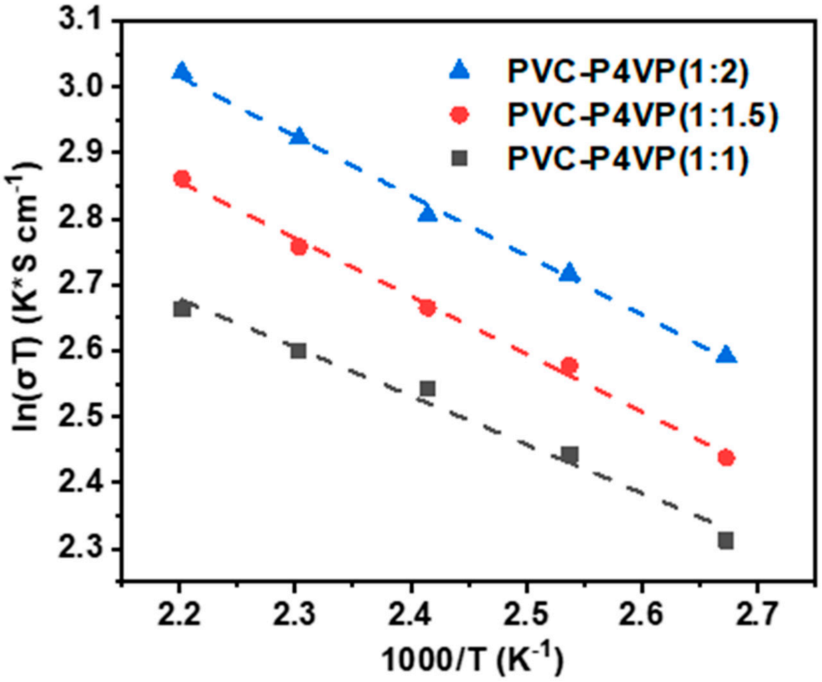

Figure S8. Temperature dependence of conductivity with PVC-P4VP membranes.

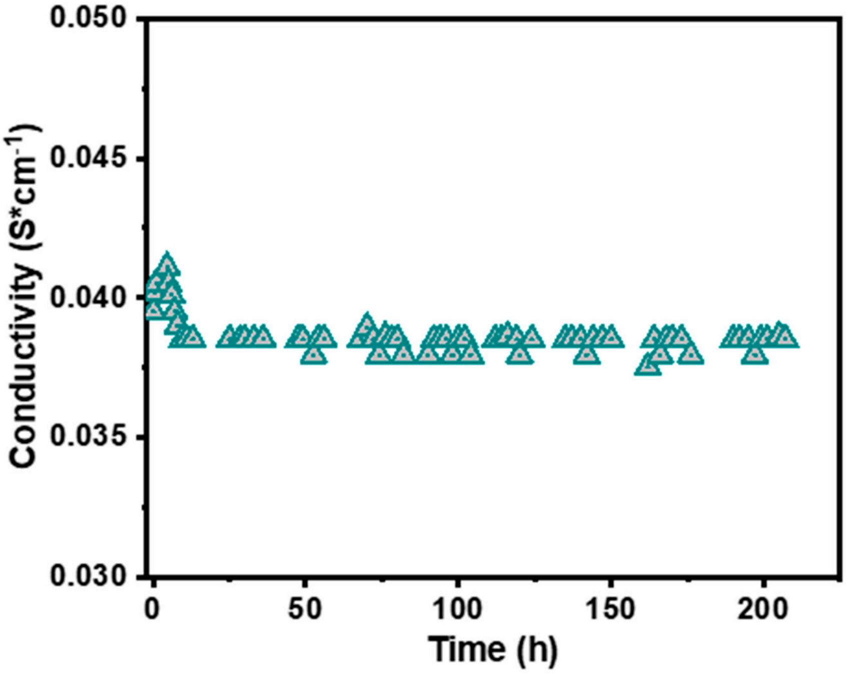

Figure S9. Stability of proton conductivity of PVC-P4VP(1:2)/PA membrane at 140°C.

Tables

Table S1. The thickness and surface area of membranes.

Membrane	Area (cm^2)	Thickness (μm)
PVC-P4VP(1:1)	0.91	230
PVC-P4VP(1:1.5)	0.95	270
PVC-P4VP(1:2)	1.08	300

Table S2. Stress-strain parameter for PVC, P4VP, PVC-P4VP(1/X) and PVC-P4VP(1/X)/PA membranes.

	**Elasticity Modulus**	**Elongation at break**	**Tensile stress at break**	**Tensile strength**
	MPa	%	MPa	MPa
P4VP	953.94	6.67	8.01	18.76
1/1	996.21	9	17.71	43.76
1/1.5	764.64	8.33	15.04	33.91
1/2	360.5	10	9.27	16.19
1/1 PA	11.02	116.05	1.49	2.54
1/1.5PA	3.93	104.55	1.3	1.63
1/2PA	1.92	94.94	0.46	1.05
PVC	1965.11	15.16	42.39	100.45
